# Effect of increased serum 25(OH)D and calcium on structure and function of post-menopausal women: a pilot study

**DOI:** 10.1007/s11657-020-00814-4

**Published:** 2020-10-03

**Authors:** H. J. Hillstrom, R. Soeters, M. Miranda, S. I. Backus, J. Hafer, M. Gibbons, I. Thaqi, M. Lenhoff, M. T. Hannan, Y. Endo, T. Sculco, J. Lane

**Affiliations:** 1 Leon Root Motion Analysis Laboratory (LRMALab), Hospital for Special Surgery (HSS), 535 East 70th Street, New York, NY USA; 2 Biomechanics Lab, Department of Kinesiology, University of Massachusetts, Totman rm.110, 30 Eastman Lane, Amherst, MA USA; 3 Institute for Aging Research, Hebrew SeniorLife, Harvard Medical School, 1200 Centre Street, Boston, MA USA; 4 Metabolic Bone Disease Service, HSS, 535 East 70th Street, New York, NY USA

**Keywords:** Recommended serum 25(OH)D levels, Muscle cross-sectional area, Postural sway, Isometric strength, Muscle fatigue, Functional task times

## Abstract

**Summary:**

The purpose was to determine if increasing serum 25(OH)D and calcium in postmenopausal women increased skeletal muscle size, strength, balance, and functional task performance while decreasing muscle fatigue. PCSA of the vastus lateralis increased and ascent of stairs time decreased after 6 months of increased serum 25(OH)D.

**Purpose:**

The Institute of Medicine recommends ≥ 20 ng/ml of serum 25-hydroxyvitamin D [25(OH)D] for bone and overall health. Serum 25(OH)D levels have been associated with physical performance, postural sway, and falls. The purpose of this study was to determine if increasing postmenopausal women’s serum 25(OH)D levels from 20–30 ng/ml to 40–50 ng/ml improved skeletal muscle size, strength, balance, and functional performance while decreasing skeletal muscle fatigue.

**Methods:**

Twenty-six post-menopausal women (60–85 years old) with baseline serum 25(OH)D levels between 20 and 30 ng/ml were recruited. Oral over-the-counter (OTC) vitamin D3 and calcium citrate were prescribed to increase subjects’ serum 25(OH)D to levels between 40 and 50 ng/ml, serum calcium levels above 9.2 mg/dl, and PTH levels below 60 pg/ml, which were confirmed at 6 and 12 weeks. Outcome measures assessed at baseline and 6 months included muscle physiological cross-sectional area (PCSA), muscle strength, postural balance, time to perform functional tasks, and muscle fatigue. Repeated measures comparisons between baseline and follow-up were performed.

**Results:**

Nineteen subjects completed the study. One individual could not afford the time commitment for the repeated measures. Three individuals did not take their vitamin D as recommended. Two subjects were lost to follow-up (lack of interest), and one did not achieve targeted serum 25(OH)D. Vastus lateralis PCSA increased (*p* = 0.007) and ascent of stair time decreased (*p* = 0.042) after 6 months of increasing serum 25(OH)D levels from 20–30 ng/ml to 40–50 ng/ml. Isometric strength was unchanged. Anterior-posterior center of pressure (COP) excursion and COP path length decreased (*p* < 0.1) albeit non-significantly, suggesting balance may improve from increased serum 25(OH)D and calcium citrate levels.

**Conclusions:**

Several measures of muscle structure and function were sensitive to elevated serum 25(OH)D and calcium levels indicating that further investigation of this phenomenon in post-menopausal women is warranted.

## Introduction

Serum 25(OH)D is widely acknowledged as the best measure of an individual’s vitamin D status. The National Institutes of Health (NIH) and Institute of Medicine (IOM) recommend ≥ 20 ng/ml of serum 25(OH)D for bone and overall health in healthy individuals (Table 1) [1, 2]. Despite this recommendation, the definition of adequate serum 25(OH)D levels varies amongst investigators (≥ 12.5 ng/ml) [3] (≥ 30 ng/ml) [4] and professional organizations ((IOM [2] (≥ 20 ng/ml), Vitamin D Task Force [5] (40–80 ng/ml), and the Endocrine Society [6] (30–100 ng/ml)).

**Table 1:** IOM vitamin D recommendations

25(OH)D*	Health status
< 12	Associated with vitamin D deficiency, leading to rickets in infants and children and osteomalacia in adults
12–20	Generally considered inadequate for bone and overall health in healthy individuals
≥ 20	Generally considered adequate for bone and overall health in healthy individuals
> 50	Emerging evidence links potential adverse effects to such high levels, particularly > 60 ng/ml

*Serum concentrations of 25(OH)D are reported in nanograms per milliliter (ng/ml)

### Purported vitamin D effects upon muscle strength, balance, and falling

Maintaining sufficient serum 25(OH)D (30–60 ng/ml) has been associated with benefits to musculoskeletal health, muscle strength [7–9], balance [10], and decreased risk of falls. Vitamin D deficiency has been associated with sarcopenia [11], reduced physical activity [8, 10–12], increased risk for falls [10], and fall-related fractures in older adults [13]. A direct link has been suggested between vitamin D and physical performance in community dwelling older adults [14]. Increasing serum 25(OH)D has also resulted in no change in physical performance in several studies [15, 16]. The disparity in conclusion is, in part, a result of variations in thresholds for adequate vitamin D, subject inclusion criteria, and outcome measurements. Still, a meta-analysis of 26 randomized controlled trials demonstrated the benefit of vitamin D along with calcium supplementation in prevention of falls in elderly women [17].

### Vitamin D toxicity

Acute toxicity from vitamin D supplementation is rare and consists principally of acute hypercalcemia, which results from doses that exceed 10,000 IU per day; associated serum levels of 25(OH)D were well above 150 ng/ml [13]. Chronically elevated 25(OH)D over 40–45 ng/ml has been associated with increased falls risk [18]. In this study, the targeted serum 25(OH)D of 40–50 ng/ml is below what is considered toxic.

These features of 25(OH)D appear to support a link between supplementation in older persons, but a gap in knowledge exists as to whether increasing baseline 25(OH)D to 40–50 ng/ml in women who are insufficient will have measurable effects on common muscle outcomes, especially so in post-menopausal women who are at higher risk for falls, fractures, and other musculoskeletal outcomes. Thus, we examined the ability to increase serum levels of 25(OH)D to 40–50 ng/ml in a sample of post-menopausal women and the 6-month effects upon key muscle and function outcomes.

The central hypothesis was that increasing serum 25(OH)D from 20–30 ng/ml to 40–50 ng/ml will increase muscle strength, reduce muscle fatigue as measured by EMG, enhance balance, and improve physical function in post-menopausal women.

## Methods

### Study population

Twenty-six post-menopausal women with baseline serum 25(OH)D levels between 20 and 30 ng/ml who were candidates for oral vitamin D supplementation were recruited for participation from the patients of a metabolic bone disease specialist (author J.L.). The inclusion criteria for enrollment were postmenopausal women (defined by absent menarche for at least 1 year), aged 60 to 85 years, able to walk 2 blocks and climb 2 flights of stairs without assistive devices (community ambulators), able to follow simple instructions, and able to perform single leg standing for 3 or more seconds. At the time of enrollment, the following serum levels were required: vitamin D levels between 20 and 30 ng/ml, calcium levels above 9.2 mg/dl, and PTH levels below 60 pg/ml. All participants had a bone mineral density measured T-score of − 1.5 or better in the spine or femur. Study participants were excluded if they had current or previous neurological diagnoses that affected ambulation, current or previous diagnosis of moderate to severe spinal deformity (i.e., scoliosis), limb length inequality greater than 2 cm, sarcoidosis, active cancer, a history of kidney stone or renal impairment, irritable bowel syndrome, past bariatric surgery, celiac sprue disease, primary hyperparathyroidism, current or previous diagnosis of metabolic bone disease other than postmenopausal osteoporosis, or received anti-osteoporotic agents with anabolic effects (e.g., estrogen, or selective estrogen receptor modulators (SERMs)), during the past 12 months. Although estrogen and SERMS have different mechanisms of actions, both drugs could affect muscle strength, we excluded individuals on agents other than bisphosphonates or teriparatide to avoid confounding variables and ensure the homogeneity of the study population. Subjects were excluded if they were in a formal lower extremity strength training program (i.e., physical therapy, personal training). One subject was excluded from the analysis since the target serum 25(OH)D level of 40–50 ng/ml was not reached at 12 weeks.

### Study design and testing protocol

**Fig. 1:**
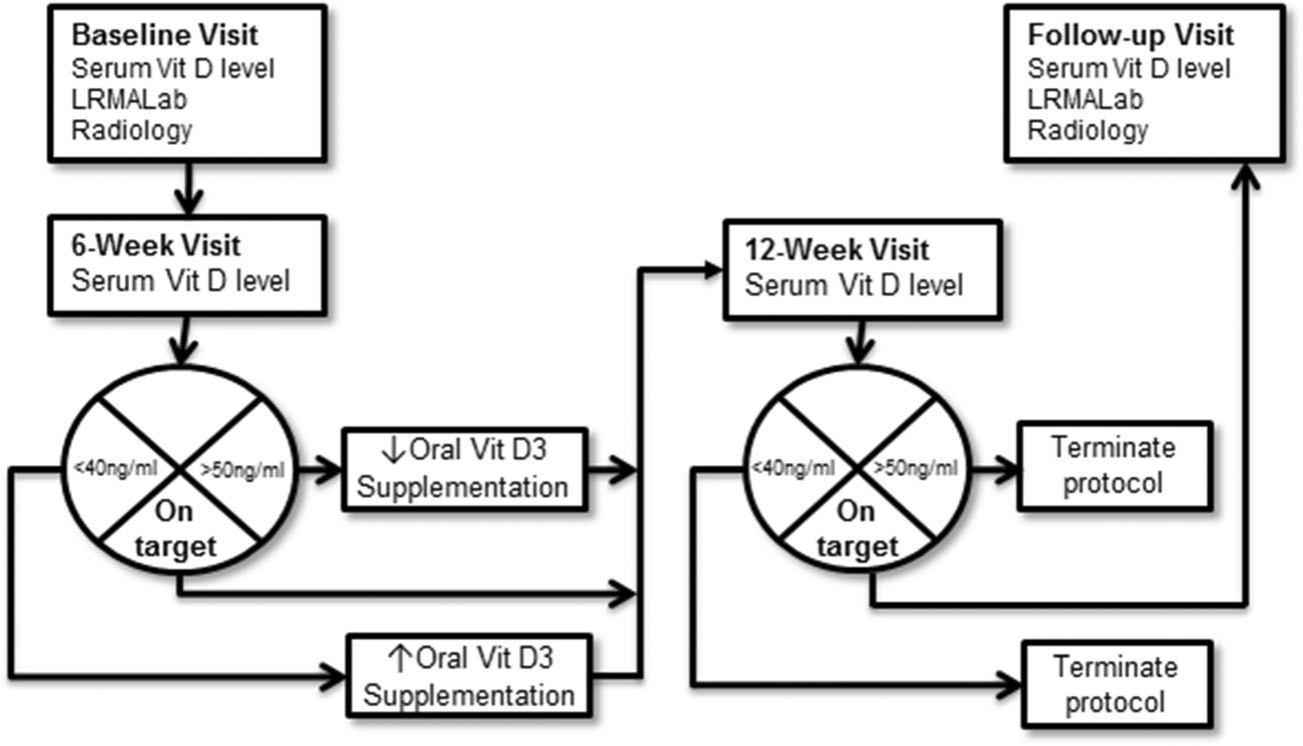
Vitamin D protocol—Baseline and 6 months after achieving target serum 25(OH)D levels

This pilot investigation was a prospective cohort design with repeated measurements comprised of baseline and 6-month follow-up once the target serum 25(OH)D levels were obtained (Fig. 1). This study received Institutional Review Board approval for human subject assurance at the Hospital for Special Surgery (HSS), and all subjects provided signed informed consent. Dual-energy X-ray absorptiometry (DEXA) scans were performed at baseline as a screening tool to ensure that lumbar spine and femoral neck T-score inclusion criteria were met. At baseline, serum 25(OH)D levels were assessed for each participant. Vitamin D3 and calcium citrate supplementation were prescribed by a metabolic bone disease specialist (author J.L.) to bring serum 25(OH)D levels to 40–50 ng/ml and serum calcium to 9.2 mg/dl or greater. Strength, balance, functional task performance, and muscle fatigue were obtained pre-vitamin D supplementation and measured in the Leon Root, MD Motion Analysis Laboratory (LRMALab) at HSS. Physiological cross-sectional area (PCSA) of relevant musculature (vastus lateralis and semimembranosus) was assessed in the Radiology Department at HSS.

Serum 25(OH)D was measured at the HSS laboratory for medicine at baseline. The Abbott Architect Immunochemistry Analyzer was used to perform the ARCHITECT 25-OH vitamin D Assay which was standardized against NIST SRM 2972 (National Institute of Standards Reference Material 2972). The performance specifications for the test were (1) the total coefficient of variation (CV < 5%) and (2) the mean bias = − 17.4% for vitamin D insufficient and − 8.9% for vitamin D supplemented subjects [19].

Serum 25(OH)D levels were repeated at 6 weeks and 12 weeks at a laboratory of convenience for the patient with adjustments in over-the-counter (OTC) vitamin D3 (cholecalciferol) dosage made if the target levels were not between 40 and 50 ng/ml. All outside laboratories are part of the network used for routine clinical practice. When target serum 25(OH)D levels were reached, follow-up testing was scheduled 6 months later. The 6-month follow-up data collection replicated all baseline measurements.

Subjects were monitored every 6 weeks as per routine clinical protocol to ensure serum 25(OH)D levels did not exceed 50 ng/ml. All patients were thoroughly screened for potential risk for vitamin D–related toxicity; no patient exceeded serum 25(OH)D levels greater than 50 ng/ml, demonstrated adverse effects, or indicated hypersensitivity.

The goal of the oral OTC vitamin D and calcium supplementation was to increase the serum 25(OH)D from 20–30 ng/ml to 40–50 ng/ml. Calcium supplementation of 500 mg to 1000 mg was typically required to achieve this goal and included as part of the clinical standard of care for this population.

### Study hypotheses

The following hypotheses were tested in the investigation:

- H1: After 6 months of oral vitamin D administration, muscle strength (isometric knee extension and flexion torque) and muscle structure (vastus lateralis and semimembranosus physiological cross-sectional area (PCSA)) will increase, while muscle fatigue (electromyography (EMG) median frequency shift) will decrease compared with the baseline as an indicator of improved muscle function.
- H2: After 6 months of oral vitamin D administration, postural sway area will decrease during both single and bipedal stances compared with the baseline as an indicator of improved balance.
- H3: After 6 months of oral vitamin D administration, functional task performance times will decrease when compared with the baseline as an indicator of improved functional performance.

### Baseline visit testing procedure

#### LRMALab

Demographics were collected at enrollment. At baseline, participants performed (1) bipedal and single leg postural stabilograms to study balance, (2) timed measures of functional tasks to assess performance, (3) maximum voluntary isometric contraction (MVIC) knee flexion and extension torque testing to assess strength, and (4) surface EMGs during a sustained sub-MVIC to determine muscle fatigue.

**Fig. 2:**
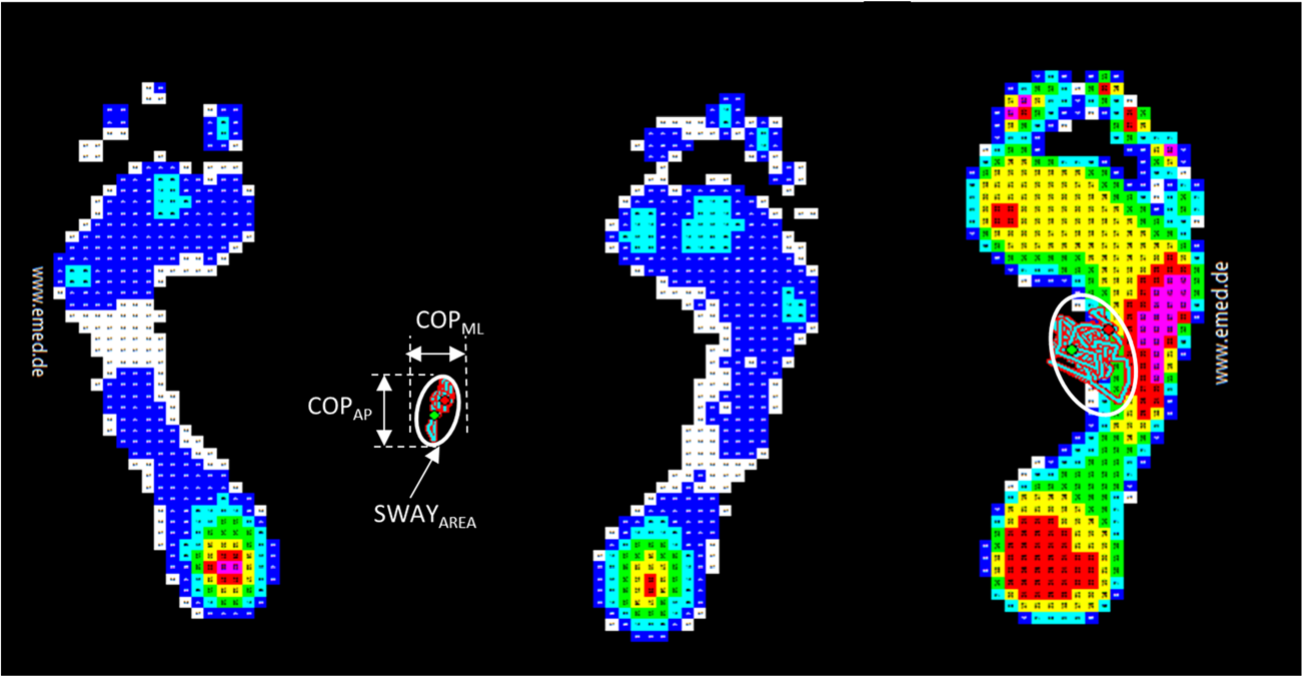
Bipedal postural stabilogram *(left)* and single leg postural stabilogram *(right)*

1. Postural stabilogram: To evaluate bipedal posture during quiet standing, bilateral plantar pressures were acquired while subjects stood with their feet at a self-selected angle and base of support upon an emed-X (Novel, Munich, Germany). With arms to both sides looking straight ahead, the subject stood for 60 s (Fig. 2, left) for three trials. For single leg postural stabilogram, subjects then stood on their self-reported dominant limb (the one they would use to kick a soccer ball) (Fig. 2, right) for 30 s. If the subject was unable to maintain postural stability for the duration of the trial, then the data was analyzed for the time that they were able to maintain stability. The center of pressure (COP) maximum excursion was determined in the anterior-posterior (COP_AP_) and medial-lateral (COP_ML_) directions. To calculate the sway area (SWAY_AREA_(cm^2^)), a 95% confidence interval ellipse was fit to the COP trajectory.

(2) Timed functional tasks: We recorded the time taken to complete each of three functional tasks: timed up and go (TUG), walking 50’ on a straight and level course at a comfortable speed, and ascent of 10 steps (rails were available if subjects needed to use them) [20]. The TUG test began with the participant seated in a chair with arms. The participant was instructed “When I say go…”, (1) stand up from the chair, (2) walk to the line on the floor (10’ from the chair) at your normal pace, (3) turn, (4) walk back to the chair at your normal pace. and (5) sit down again.
(3) Dynamometry: bilateral isometric strength testing of the knee extensor and flexor muscles was performed using an isokinetic dynamometer (Biodex System 4, Biodex Medical Systems, Shirley, NY). The subject sat on the testing bench with hips in 80° of flexion; back supported in a comfortable position; and pelvis stabilized with a waist belt according to the standard operating procedures for knee isometric testing. An additional padded stabilization strap was placed over the anterior aspect of the thigh. Isometric testing was completed at 30° and 60° of knee flexion. Three 5-s trials of maximal effort were collected with a 10-s rest between trials, a 20-s rest between positions, and a 2-min rest period between sides. MVIC strength of the knee extensors and flexors was defined as the peak torque (Nm) across the three trials.
(4) EMG testing: Before isokinetic dynamometry, surface EMG electrodes were applied according to standard clinical testing protocol as follows: the skin was prepared with isopropyl alcohol to reduce impedance. EMG surface electrode preamplifiers were placed on the vastus lateralis and vastus medialis according to SENIAM guidelines and confirmed on a real-time digital display [21]. EMG data was acquired during a MVIC while making the dynamometry measurements. After a 2-min rest following the MVIC, and in the same position, seated on the isokinetic dynamometer, subjects performed a sustained isometric knee extension contraction at 50% of MVIC. Subjects were given visual feedback (a real-time bar graph on a computer monitor with a target line) and verbal cues from the tester (i.e., “kick harder,” “hold it steady at that level”) to help them maintain a knee torque that was 50% MVIC [22]. This contraction was maintained for 1 min. If the subject could not sustain the 50% MVIC for 1 min, then their best effort was recorded for the duration of the trial. The raw EMG data was divided into 250 ms time segments. Each segment was transformed into the frequency domain and the median power frequency for each segment calculated. The median power frequency values were plotted versus time and a linear regression calculated. The steeper the line (i.e., more negative the slope), the greater the rate of fatigue.

#### Radiology department

DXA measurements were taken prior to enrollment as part of the standard of care of the metabolic bone disease specialist (J.L.) who recruited each subject for this study. DXA measurements served as a screening tool to ensure that all participants had a bone mineral density measured T-score of − 1.5 or better in the spine or femur.

##### Ultrasound

A board-certified radiologist at HSS with subspecialty expertise in musculoskeletal ultrasound (author Y.E.) assessed each subject’s vastus lateralis and semimembranosus PCSA (cm^2^) with ultrasound using an iU22 scanner (Philips Healthcare) with a 9 MHz linear transducer [23]. First, with the subject seated and knees flexed to 90°, the PCSA of the right and left vastus lateralis was measured after visually approximating the mid femur (mid-thigh) level. This was repeated twice and the average of the three PCSA measurements was used to minimize the subjectivity associated with identifying the mid femur location. Next, with the subject prone and hips and knees extended, the PCSA of the bilateral semimembranosus was measured after visually approximating the mid femur level. Again, this was repeated twice and the average of the three measurements was recorded for each side. All ultrasound measurements were scheduled to immediately follow LRMALab testing and conducted within the department of radiology of HSS.

#### Oral vitamin D supplementation

After baseline testing was performed, the subject began oral vitamin D3 supplementation to achieve target serum 25(OH)D levels between 40 and 50 ng/ml. Vitamin D was prescribed by our metabolic bone disease specialist (author J.L.). If the patient’s serum 25(OH)D was at 20 ng/ml, they were prescribed 4000 IU. Initial dosage was individually determined based upon the subject’s baseline serum 25(OH)D levels. Oral vitamin D3 was dosed at either 1000, 2000, 3000, or 4000 IU daily, standard dosing units. Dosage was ≤ 4000 IU/day for 6 weeks to achieve target serum 25(OH)D levels between 40 and 50 ng/ml. At 6 weeks the patient’s serum 25(OH)D level was retested. In general, 2000 IU to 3000 IU per day was required to maintain a level between 40 and 50 ng/ml (i.e., the target range) for 6 months. Subjects received calcium citrate supplementation from standard over-the-counter sources. Patients were prescribed 500 mg daily if dairy was part of their diet and 1000 mg if not. Calcium citrate dosage was adjusted to achieve and maintain serum calcium levels above 9.2 mg/dl and PTH levels below 60 pg/ml. Serum PTH level was monitored.

Six weeks after initiating vitamin D and calcium supplementation, each subject was required to obtain another serum analysis to adjust their supplementation doses. Target levels of serum 25(OH)D (40–50 ng/ml), calcium above 9.2 mg/dl, and PTH below 60 pg/mL were maintained with appropriately adjusted doses. Laboratory results were ordered and archived by the Metabolic Bone Disease Clinic of HSS. Subjects who did not achieve the target 25(OH)D level by the 12-week blood test were terminated. Subjects were informed regarding any required changes in vitamin D3 dose by phone and by study physicians. The last periodic check was performed at 3 months since vitamin D has a half-life from 4 to 8 weeks. Note that only one subject did not achieve the target 25(OH)D level by the 12-week blood test.

### Follow-up visit—LRMALab and radiology department

Six months after each subject had reached the target serum 25(OH)D level of 40–50 ng/ml, follow-up visits at the LRMALab and the radiology departments of HSS were performed. The same testing protocol used at baseline was repeated.

### Statistical analysis

The primary outcomes were as follows: (1) strength *(peak torque during MVIC) defined as the maximum torque during isometric contraction in any one of the three trials of knee extension and flexion as measured on the Biodex dynamometer)*; (2) muscle area *(vastus lateralis and semimembranosus PCSA (cm*^*2*^*), defined as an ultrasound based measure of the muscle diameter at multiple levels along the length of the thigh, serving as an estimate of muscle force)*; (3) muscle fatigue *(median frequency of the vastus lateralis and semimembranosus muscle EMGs during a 50% MVIC defined as the frequency where half the power is above and half the power is below)*; (4) balance (postural sway area (SWAY_AREA_) during a 60-s (30 s) postural stabilogram in bipedal (single leg) stance defined as the area of a best elliptical fit to center of pressure (COP) sway at the 95% confidence interval); and (5) functional performance *(the time in seconds taken to complete each of three functional tasks (rising from a 42-cm chair height, walking 50’ at a comfortable speed, and ascent and descent of 10 steps)).*

The secondary outcomes were (1) balance *(anterior-posterior and medial-lateral maximum (COP*_*AP,*_ *COP*_*ML*_*) sway for single and bipedal stance, single leg stance time).*

Data analysis included descriptive statistics (means, standard deviations, coefficient of variation) for all primary and secondary outcome variables collected at baseline and after 6 months of achieving target 25(OH)D levels. Each outcome variable was tested for normality (Wilks-Shapiro, Q-Q plots) and homogeneity of variance (Levine’s test). Comparative statistics were employed to test each hypothesis. Primary and secondary outcome variables were compared pre- and post-treatment protocol using a generalized estimation equation (GEE)–based model for bilateral data to account for potential dependence between limbs or repeated measures analysis of covariance (ANCOVA) across time (baseline and 6-month follow-up). A *p* value ≤ 0.05 was set for statistical significance. A *p* value ≤ 0.10 was set for identifying parameters of interest for future investigation (borderline statistical significance). All data analysis was conducted by using SPSS software (version 19.0).

## Results

### Study population

Twenty-six post-menopausal women met inclusion/exclusion criteria and were enrolled in the study. Seven participants were lost to follow-up (unrelated medical conditions, did not answer phone for scheduling, missed follow-up appointment, and could not reschedule), and one subject was unable to achieve the target vitamin D level by 12 weeks. Nineteen subjects completed baseline and follow-up testing. Of these participants, age (mean ± SD) was 70.1 ± 9.2 years, body weight was 58.8 ± 10.9 kg, height was 160.7 ± 6.1 cm, and body mass index (BMI) of 22.9 ± 4.6 kg/m^2^. The T-scores at the lumbar spine was − 1.18 ± 1.6 and at femoral neck was − 1.87 ± 0.8. Seventeen participants were white, 1 black, and 1 Asian with all but three participants being right limb dominant. One subject was unable to obtain the 6-month ultrasound measures. Table 2 summarizes the effect of elevating serum 25(OH)D levels from 20–30 ng/ml to 40–50 ng/ml upon differences in muscle structure, strength, and fatigue. The PCSA (cm^2^) significantly increased (*p* < 0.05) for the vastus lateralis but not for the semimembranosus after 6 months of elevated serum 25(OH)D levels. Muscle strength and fatigue were unchanged.

**Table 2:** Baseline and follow-up measures of muscle structure, fatigue, and strength

*N* = 19	N_sub_	N_limbs_	V_1_	SD V_1_	V_2_	SD V_2_	χ^2^	*p* value	^*^Power	^^^*N (α < 0.05; p > 0.8)*
PCSA (cm^2^)										
Vastus lateralis (cm^2^)	*18*	*36*	*10.9*	*3.4*	*12.4*	*3.7*	*7.40*	*0.007*	*0.82*	*37*
Semimembranosus (cm^2^)	18	36	7.0	1.7	7.1	1.3	0.074	0.785	*0.103*	*1467*
EMG—muscle fatigue (Hz/s)										
Quadriceps slope (Hz/s)	19	38	− 0.1	0.1	− 0.1	0.1	1.16	0.281	*0.05*	
Hamstring slope (Hz/s)	19	38	− 0.2	0.2	− 0.2	0.3	0.092	0.761	*0.05*	
Isometric strength (Nm)										
Knee extension (Nm)	19	38	108.6	30.4	105.8	27.0	0.268	0.605	*0.145*	*658*
Knee flexion (Nm)	19	38	52.9	15.6	51.2	11.1	0.68	0.410	*0.18*	*416*

^*^Power 1-β achieved post hoc, ^*^*^*N* sample size required for effect size achieved, and *α < 0.05; p > 0.8*

Table 3 lists the effect of elevating serum 25(OH)D levels upon balance, functional tasks, and endurance between baseline (Visit_1_) and follow-up (Visit_2_). During bipedal posture, anterior-posterior (AP) center of pressure (COP) excursion and center of pressure path length were different (*p* < 0.1) over follow-up. One leg postural stabilogram parameters over 6-months were not significantly changed following increased serum 25(OH)D. The coefficient of variation (S/X) in bipedal elliptical sway reduced from 120% to 54% and in one leg elliptical sway from 92% to 65%. These changes in postural sway and COP excursion are indicative of improved balance. The 10-stair ascent time was significantly reduced after increased serum 25(OH)D levels. The TUG and 50’ walk times were not significantly changed after 6 months. Endurance as measured by single limb stance times was not significantly changed as well.

**Table 3:** summary of vitamin D study effects upon balance, activities of daily living, and endurance

*N* = 19	N	V_1_	SD V_1_	V_2_	SD V_2_	F	*p* value	^*^Power	^^^*N (α < 0.05; P > 0.8)*
Balance—bipedal									
Elliptical sway area (cm^2^)	19	3.0	3.6	1.9	1.0	1.84	0.191	0.416	55
AP excursion (cm)	*19*	*2.3*	*1.1*	*1.7*	*1.0*	*3.403*	*0.082*	*0.771*	*21*
Path length (cm)	*19*	*60.4*	*22.5*	*52.4*	*12.7*	*3.617*	*0.073*	*0.528*	*39*
Balance—single leg									
Elliptical sway area (cm^2^)	19	24.2	22.3	19.4	12.7	1.919	0.183	0.272	103
AP excursion (cm)	19	7.0	5.0	6.1	3.1	0.906	0.354	0.217	148
Path length (cm)	19	78.2	56.3	83.1	45.2	0.240	0.629	0.106	689
Single limb stance time (sec)	19	15.7	9.1	16.9	9.7	0.644	0.433	0.133	382
Functional tasks (sec)									
Timed up and go (TUG) (sec)	19	13.9	2.5	13.9	2.8	0.00	0.994	0.05	
50’ walk (sec)	19	12.6	2.3	12.8	2.4	0.175	0.681	0.098	857
10 stair ascent (sec)	*19*	*7.0*	*3.0*	*5.8*	*1.2*	*4.773*	*0.042*	0.609	31

^*^Power 1-β achieved post hoc, ^*^*^*N* sample size required for effect size achieved, and *α < 0.05; P > 0.8*

A post hoc power analysis was performed. The achieved power based upon effect sizes observed for each outcome measure is shown in Tables 2 and 3. The sample size required for the observed effect size, α < 0.05, and power > 0.8 was computed for each outcome measure. Seven of sixteen outcome measures would be properly powered with an *N* = 148.

## Discussion

Physiological cross-sectional area (PCSA), a measure of gross muscle structure, increased in the vastus lateralis after increasing serum 25(OH)D levels from 20–30 ng/ml to 40–50 ng/ml. Although this did not translate into an increase in knee extension moment, there was a functional improvement in the time required to ascend 10 stairs. This change in muscle structure provided partial support for hypothesis 1. Muscle fatigue and bipedal and one leg postural stabilogram parameters did not significantly change after increased levels of serum 25(OH)D. In this small group, AP COP excursion and path length demonstrated a trend for reduction and may hold promise in a larger sample. Coefficient of variation for elliptical SWAY_AREA_ during bipedal and one leg postural sway was also reduced, so hypothesis 2 was partially supported. Time for ascent of 10 stairs was significantly reduced and provided partial support for hypothesis 3 of this study.

Our study focused upon increasing serum 25(OH)D from what the IOM considers adequate (20–30 ng/ml) to 40–50 ng/ml. Results from our study differ compared with the literature based upon serum 25(OH)D levels studied. For example, Cangussu et al, reported post-menopausal women receiving vitamin D3 orally had a significant increase in lower limb muscle strength (25.3%) as measured by the chair rising test while those in the placebo group lost lean muscle mass [7]. This study raised serum 25(OH)D in the treatment group from 15.0 to 27.5 ng/ml, which was lower than the baseline level included in our current study. Their inclusion of individuals with a lower baseline of serum 25(OH)D may have resulted in greater room for lower limb muscle strength improvement compared with our study.

Iolascon et al (2015) evaluated 80 post-menopausal women [24] and found that those women with serum 25(OH)D levels > 30 ng/ml had increased handgrip strength, knee extension strength, 4-m gait speed, and improved short physical performance battery compared with the hypovitaminosis group (serum 25(OH)D levels < 30 ng/ml) [24]. Although in our subjects, knee extension strength was unchanged, we were able to demonstrate a larger PCSA for the vastus lateralis and improved 10 step stair ascent time suggesting improved quadriceps structure and function.

Mathei et al (2013) studied 367 individuals over 80 years old to determine the prevalence of vitamin D deficiency (20 ng/ml), insufficiency (10–19 ng/ml), and severe insufficiency (< 10 ng/ml) and found no relation between balance, gait speed, and grip strength [25]. It is possible that once vitamin D levels were inadequate, stratification across levels of physical performance was more difficult to observe [25]. Taken together, the differences in protocol designs, age of subjects, and stratification by different serum 25(OH)D levels may in part explain the differences in outcomes reported in the literature compared with our study.

In a 2017 meta-analysis (*n* = 2866), no change in strength due to higher 25(OH)D levels was reported but the TUG significantly reduced (improved) [26]. Similarly, the subjects in our study had no improvement in strength following higher 25(OH)D levels. Although the TUG was unchanged, another functional performance measure (stair ascent time) improved. Perhaps this was related to the need for a combination of increased strength and balance to complete the higher demand task of stairs rather than the requirements to perform quiet standing or TUG test.

In a 4-week study of vitamin D supplementation in post-menopausal women, balance significantly improved [27]. Saito et al. randomly assigned 50 post-menopausal women with osteoporosis to receive vitamin D supplement and bisphosphonates or bisphosphonates only [28] and found, at 6 months, no changes in the control group, while the vitamin D group’s back extensor and iliopsoas muscle strength as well as functional performance (TUG test and dynamic sitting balance) significantly increased. We found that AP COP excursion and the COP path length showed a trend for reduction after 6-months of elevated serum 25(OH)D (*p* < 0.1), suggesting balance may improve after vitamin D supplementation.

Furthermore, low serum 25(OH)D levels (< 10 ng/ml) have been linked to a higher risk of repeated falling [14, 29–31]. Vitamin D supplementation in vitamin D–deficient older adults results in a reduction in falls [12, 31–35]. Meta-analysis of five randomized controlled trials has shown that vitamin D supplementation of ≥ 700 IU resulted in decreased risk of falls by 22% [36]. In addition, meta-analysis of 26 randomized controlled trials demonstrated the benefit of vitamin D along with calcium supplementation in prevention of falls in elderly women [17].

Increased body sway, higher risk of falls, and fall-related fractures are linked with hypovitaminosis D [37]. When vitamin D–deficient (≤ 12 ng/ml) individuals with a history of falls were treated with intramuscular injection of 600,000 IU of vitamin D_2_, subjects showed improved performance, reaction time, and balance, but not muscle strength after 6 months [32]. Although evidence demonstrates a positive association between serum 25(OH)D and balance along with muscle fiber distribution and strength, the minimum requirement for improving these parameters and prevention of falls remains controversial. The clinical implications of enhanced balance and reduced muscle fatigue may decrease the number of falls and fractures in these individuals.

There were several strengths and limitations to our study. The strengths included are as follows: (1) participants with low serum 25(OH)D were recruited and confirmed with a higher 25(OH)D level following supplementation, and (2) outcomes were based upon objective measurements. The confirmed higher 25(OH)D level after supplementation permitted the objective assessment of changes in muscle strength, structure, and function as well as postural sway and functional performance from recommended IOM levels of 20–30 ng/ml to the target levels of 40–50 ng/ml.

The limitations included (1) lack of an independent, randomized, untreated control group and a small sample size (*n* = 19), with only a single follow-up at 6 months, (2) several different labs were used for serum 25(OH)D testing which incorporated different methods for this assay, and (3) 25(OH)D supplementation was given with calcium which may have confounded effects. Future larger randomized controlled trial designs should benefit from the data acquired in this study to generate effect sizes for sample size calculations. Short- and/or long-term follow-up changes in response to higher 25(OH)D levels may have been missed at a single 6-month follow-up visit. The 20% loss to follow-up could be minimized in future studies with interim visits over the follow-up period.

Several different laboratories were used for follow-up serum 25(OH)D testing which incorporated different methods for this assay. This could have led to an over-estimate or under-estimate in serum 25(OH)D levels. Specific to this study, it was possible that we included someone in the 20–30 ng/ml baseline group or the elevated 40–50 ng/ml group that was outside of the target range. Future studies should try to standardize serum 25(OH)D testing to one laboratory, store an additional sample for future testing of each subject at each visit, perform post-hoc standardization analyses based upon standardized reference materials, and, if possible, participate in the CDC lab standardization program for this assay.

Several investigators have established that vitamin D_3_ [37, 38] or vitamin D_2_ supplementation along with calcium is more effective in preventing falls as compared with calcium alone. This finding has been contradicted in a study where falls were best predicted by sarcopenia and lean muscle mass [39]. In the setting of sarcopenic obesity, a combination of obesity with low skeletal muscle mass and correction of vitamin D deficiency had a positive role on muscle mass [40].

## Conclusions

The clinical significance of the preliminary findings includes the potential to increase PCSA of the vastus lateralis (muscle structure) and enhance performance while ascending stairs (function) and potentially reducing AP COP excursion and COP path length (measures of balance). Vitamin D supplementation is relatively easy to administer and generally has good compliance due to its well-accepted benefit for managing osteoporosis. Finally, vitamin D supplementation is relatively of low cost which obviates many financial barriers to treatment. The potential for having a relatively low cost means to enhance structure, function, and balance in post-menopausal women that could potentially lower the incidence of falls and concomitant fracture makes this area important for further investigation.

The use of vitamin D supplementation to improve muscle structure, muscle strength, balance, and functional task performance remains controversial. The preliminary evidence from this pilot study suggests that it may be possible to augment muscle structure, functional performance, and potentially balance through increasing serum 25(OH)D to levels between 40 and 50 ng/ml. A clinical trial that incorporates these objective outcome measures with a larger sample size, randomized treatment assignment, and a control group would appear to be warranted.
