# Correction to: Effect of increased serum 25(OH)D and calcium on structure and function of post-menopausal women: a pilot study

**DOI:** 10.1007/s11657-020-00875-5

**Published:** 2021-01-28

**Authors:** H. J. Hillstrom, R. Soeters, M. Miranda, S. I. Backus, J. Hafer, M. Gibbons, I. Thaqi, M. Lenhoff, M. T. Hannan, Y. Endo, T. Sculco, J. Lane

**Affiliations:** 1 Leon Root Motion Analysis Laboratory (LRMALab), Hospital for Special Surgery (HSS), 535 East 70th Street, New York, NY USA; 2 Biomechanics Lab, Department of Kinesiology, University of Massachusetts, Totman rm.110, 30 Eastman Lane, Amherst, MA USA; 3 Institute for Aging Research, Hebrew SeniorLife, Harvard Medical School, 1200 Centre Street, Boston, MA USA; 4 Metabolic Bone Disease Service, HSS, 535 East 70th Street, New York, NY USA

**Correction to: Archives of Osteoporosis (2020) 15: 154**

10.1007/s11657-020-00814-4

The authors were made aware of the misuse of references and erroneous work by Sato and colleagues in our paper. Therefore, the reference as well as any citation of the reference has been removed. This erratum does not affect the findings in our research paper. The authors and the journal regret the error.

We wish to thank Jose Moran et al. for bringing this to our attention.

